# No evidence for reduced Simon cost in elderly bilinguals and bidialectals

**DOI:** 10.1080/20445911.2014.929580

**Published:** 2014-06-26

**Authors:** Neil W. Kirk, Linda Fiala, Kenneth C. Scott-Brown, Vera Kempe

**Affiliations:** ^a^ Division of Psychology, Abertay University, Bell Street, DundeeDD1 1HG, UK

**Keywords:** Bidialectism, Bilingualism, Inhibitory control, Simon task

## Abstract

We explored whether a bilingual advantage in executive control is associated with differences in cultural and ethnic background associated with the bilinguals' immigrant status, and whether dialect use in monolinguals can also incur such an advantage. Performance on the Simon task in older non-immigrant (Gaelic-English) and immigrant (Bengali, Gujarati, Hindi, Malay, Punjabi, Urdu-English) bilinguals was compared with three groups of older monolingual English speakers, who were either monodialectal users of the same English variety as the bilinguals or were bidialectal users of a local variety of Scots. Results showed no group differences in overall reaction times as well as in the Simon effect thus providing no evidence that an executive control advantage is related to differences in cultural and ethnic background as was found for immigrant compared to non-immigrant bilinguals, nor that executive control may be improved by use of dialect. We suggest the role of interactional contexts and bilingual literacy as potential explanations for inconsistent findings of a bilingual advantage in executive control.

Regular use of two or more languages requires individuals to inhibit one language when using the other, to select the appropriate linguistic setting and individual words in the target language (Green, 1998; Hilchey & Klein, 2011). Habitual inhibition of one language in bilinguals is assumed to lead to transfer of improved executive control to non-linguistic domains. This bilingual advantage, demonstrated in a number of studies (for a review see Bialystok, Craik, Green, & Gollan, 2009), appears to be most pronounced in participants in whom cognitive processes do not operate at peak speed such as young children and older adults (Bialystok, Martin, & Viswanathan, 2005). In particular, in older bilinguals, lifelong frequent use of two languages may provide the practice needed to improve executive functioning, which may help to maintain cognitive flexibility later in life (Bialystok, Craik, Klein, & Viswanathan, 2004).

Because random assignment is not possible in quasi-experimental studies comparing bilinguals with monolinguals, there remains the possibility that bilingualism covaries with other factors that can also affect executive functioning (Hilchey & Klein, 2011), such as socio-economic status (SES; Morton & Harper, 2007) and immigrant status, which is associated with differences in cultural and ethnic background. Even when bilinguals and monolinguals are matched in SES, it is often impossible to match them also in ethnic and cultural background. Consider the studies that examine older participants: In Bialystok et al. (2004), the monolinguals resided in North America whereas the majority of bilinguals resided in India. Thus, although the bilinguals were non-immigrants in their country of residence, they clearly differed in cultural and ethnic background from the monolinguals. In Bialystok, Craik, and Luk (2008), 20 out of 24 bilinguals were immigrants from ethnical and cultural backgrounds who had arrived in North America as children or adolescents. Salvatierra and Rosselli (2010) and Schroeder and Marian (2012) do not explicitly report immigrant status, yet the ages of English acquisition of their bilinguals suggest that they too were predominantly first-generation immigrants differing in ethnic and cultural background from the monolinguals. Thus, the specific bilingual populations studied so far make it difficult to disentangle bilingualism from other potential factors that may be linked to differences in executive functioning.

An association of ethnic and cultural background with executive functioning (Sabbagh, Xu, Carlson, Moses, & Lee, 2006) can be attributed to culture-specific parenting attitudes or educational and leisure practices which promote exposure to activities that require executive control, such as musical training (Bialystok, 2011), playing of video games (Green & Bavelier, 2003) and other, as of yet unstudied pursuits. Moreover, genetic effects may also contribute to ethnic differences in executive functioning. For example, population-genetic studies show that the prevalence of the 7-repeat allele of the dopamine receptor gene (DRD4) is markedly lower in East and South-East Asia compared to North America (Chang, Kidd, Livak, Pakstis, & Kidd, 1996). This allele has been associated with attention-deficit hyperactivity disorder (ADHD; Faraone, Doyle, Mick, & Biederman, 2001), which, in turn, often manifests itself in poor executive functioning (Schachar, Tannock, Marriott, & Logan, 1995). To examine these potentially confounding effects, the present study aimed to compare executive control in older immigrant and non-immigrant bilinguals. The immigrant bilinguals were speakers of English and a language spoken in Asia and differed in ethnic and cultural background from the monolinguals. The non-immigrant bilinguals were British speakers of Gaelic, an indigenous Celtic minority language used by about 58,000 individuals residing mainly in the West of Scotland. Since Gaelic-medium schooling was abolished in 1872 and reintroduced only in 2006, these participants had acquired Gaelic in early childhood before being introduced to English in school and tended to use Gaelic in the home and in the local bilingual community. Crucially, in terms of ethnic background, cultural attitudes and values, educational practices, leisure activities, media exposure, socio-economic and immigrant status, Gaelic-English bilinguals do not differ from British English monolinguals. If bilingualism, rather than ethnic or cultural background, is linked to superior executive control, then both bilingual groups should exhibit an advantage compared to the monolinguals.

A bilingual advantage in executive control may also be attenuated or aggravated by factors operating within monolinguals, such as using different dialectal varieties of a language. To date, little is known about the cognitive demands imposed by dialect use. Even though dialects are considered to be mutually intelligible, they still require management of competing phonetic and lexical variants. Compared to the relatively moderate differences between regional and social varieties of English in the USA and Canada, the countries from which the majority of monolingual controls have been recruited so far, Britain has considerable dialectal diversity: Speakers of British English are routinely exposed to many distinct varieties of English (e.g. Scots, Scouse, Geordie, etc.) in addition to the standard variety. Specifically, in Scotland, 85% of the population report using one of the local varieties of the Scots dialect to varying degrees (Scottish Government Social Research, 2010) in addition to Standard Scottish English (SSE). Local varieties of Scots differ considerably from SSE in phonetic, lexical and even some syntactic features (Smith & Durham, 2012). As a result, bidialectal speakers must be able to: monitor continuously who can or cannot be addressed in Scots, choose appropriate articulatory settings and inhibit competing phonetic and lexical variants of the language variety not currently in use. If use of multiple dialects incurs executive control benefits similar to those observed in bilinguals, then differences in executive control between bidialectal monolinguals and bilinguals might be attenuated. It is, therefore, important to control dialect use in monolinguals. To this end, we tested three monolingual control groups: (1) *bidialectal* speakers who reported switching routinely between SSE and Dundonian, a local variety of Scots spoken in North-Eastern Central Scotland, (2) *monodialectal* speakers of SSE, the English variety spoken by the Gaelic-English bilinguals, who resided in the same locale as the bidialectals, but reported not using Dundonian Scots despite having full comprehension of it and (3) *monolingual* speakers of Anglo-English, the same variety spoken in the South of England by the majority of the Asian Language-English bilinguals, and to whom Scots was unfamiliar and often unintelligible. We use the label *monodialectal* to refer to those monolingual participants who shared a geographical and cultural background with the *bidialectal* participants; however, it should be noted that these monolinguals also represent the most appropriate control group for the Gaelic-English bilinguals.

## METHOD

We examined inhibitory control using the Simon task as employed in Experiment 1 of Bialystok et al. (2004); the experiment that provided the very first evidence for an inhibitory control advantage in older bilinguals. The Simon task requires participants to inhibit a pre-potent spatial cue when responding to the colour of a stimulus. If an inhibitory control advantage arises from routine use of two languages but not dialects, regardless of ethnic and cultural background, then both bilingual groups should display a smaller Simon effect compared to the bidialectal, monodialectal and monolingual groups. If regular use of dialect also results in an inhibitory control advantage, then bidialectals too should exhibit a smaller Simon effect compared to monodialectals and monolinguals. Finally, if differences in cultural and ethnic background associated with immigrant status are related to an inhibitory control advantage, then only the Asian Language-English bilingual group should exhibit a smaller Simon effect. We measured verbal intelligence to ensure equal English proficiency between the groups, as well as non-verbal intelligence and SES to rule out these alternative sources for group differences in inhibitory control.

### Participants

Eighty older adults (mean age = 70.8 years, *SD* = 7.5 years, range = 60.0–89.0 years) participated in the experiment. Sixteen bilingual participants (six men) were speakers of Gaelic and SSE. Sixteen bilingual participants (10 men) were speakers of English and either Bengali, Gujarati, Hindi, Malay, Punjabi or Urdu who had immigrated to the UK before 35 years of age. Sixteen bidialectal participants (seven men) were speakers and regular users of both SSE and Dundonian Scots. Sixteen monodialectal participants (five men) were monolinguals speakers of SSE who had regular exposure to and could comprehend (but did not use) Dundonian Scots. Finally, 16 monolingual participants (six men) were speakers of Anglo-English spoken in the South of England. The monodialectal and bidialectal participants were recruited from the Dundee area, the Gaelic-SSE bilinguals were recruited from the Western Isles and the West coast of Scotland, the English monolinguals were recruited from different parts of England and Scotland (all but one had not lived in Scotland for any considerable length of time and were either visitors or had recently retired to the area). The Asian language bilinguals were recruited from London and Dundee, and their age of arrival in the UK (*M* = 26.6 years, *SD* = 4.8 years, range = 14.0–35.0 years) was similar to the 21.5 years reported for the bilinguals in Salvatierra and Rosselli (2010).

The Background Questionnaires (described below) revealed that the bilinguals' daily use of their non-English language(s) and the bidialectals' use of Dundonian Scots ranged between 30% and 70% of time. The monodialectals reported less than 25% use of Dundonian Scots. In addition to the 80 participants included in the final analysis, a number of other participants were tested but excluded for various reasons: Three participants reported predominantly using Dundonian Scots and, as it proved impossible to recruit further monodialectal speakers of this type, were excluded from the study. Nine bilinguals reported percentages of English use outside the 30–70% range. Two participants were excluded for having extremely low English proficiency as measured in the Vocabulary subscale of the WASI. Five participants were excluded for having an age of arrival in the UK greater than 40. Four participants failed to perform the Simon task correctly, and data for one participant were not recorded due to equipment malfunction.

### Materials

##### Background Questionnaires

One background questionnaire inquired about the participants' educational background, the occupations they had held throughout their working lives, as well as daily usage of the different varieties of English and of other foreign languages; Scottish participants were also asked about their use of varieties of Scots.

Bilinguals and bidialectals additionally received modified versions of the Language Experience and Proficiency Questionnaire (LEAP-Q; Marian, Blumenfeld, & Kaushanskaya, 2007), a questionnaire designed to determine bilingual language status through proficiency self-ratings that has been validated using behavioural measures of language proficiency. The LEAP-Q was adapted for use with dialect speakers by asking to what extent participants were fluent in one or two varieties, e.g. SSE and Dundonian Scots, and the age at which they became fluent.

##### Wechsler Abbreviated Scale of Intelligence (WASI)

Two subscales of the WASI were used to determine participants' verbal and non-verbal IQ. The Vocabulary subscale tested participants' verbal reasoning ability and ability to give definitions of words. The Matrix Reasoning subscale contained visuo-spatial patterns designed to measure abstract non-verbal reasoning ability. Participants' raw scores were converted to *t*-scores, which are normalised for each age range.

##### Simon task

We used the same procedure as Experiment 1 in Bialystok et al. (2004). Participants were presented with red and blue squares, half of which appeared on the left side of the screen and half on the right. Participants were asked to press the “1” key on the left or the “0” key on the right of the keyboard, depending on the colour of the square. The keys were marked with white stickers on the keyboard. In congruent trials, the response key associated with the colour of the square was located on the same side as the square. In incongruent trials, the response key was located on the opposite side requiring participants to inhibit the spatial cue. The reaction time difference between incongruent and congruent trials is considered to be a measure of inhibitory control. Participants were given 4 congruent and 4 incongruent practice trials with feedback before moving on to the 28 critical trials (7 each of congruent red, congruent blue, incongruent red and incongruent blue) presented without feedback.

### Procedure

Participants were first given the Background Questionnaire followed by the LEAP-Q, if appropriate, before completing the Vocabulary and Matrix Reasoning subscale of the WASI. Finally, the Simon task was administered, with presentation controlled by Eprime (Psychology Software Tools). Participants first saw a fixation cross in the middle of the screen for 800 ms, followed by an interval of 250 ms. Colour assignment to key location was counterbalanced across participants. Then, a red or a blue square appeared either to the left or the right of the screen, subtending five degrees of visual angle. The squares were visible for 1,000 ms if there was no response. Timing began with the onset of the stimulus and was terminated with the response. The next item started after a 500 ms inter-stimulus interval. The experiment began with eight practice trials for which participants' received feedback, followed by randomised presentation of 28 critical trials presented without feedback.

## RESULTS

Results for the linguistic, cognitive and demographic variables as well as the reaction times (RTs) and error rates for the Simon task of the five participant groups are given in Table 1.

**TABLE 1:** Means and standard deviations (in parentheses) for linguistic, demographic and cognitive measures

	Monolinguals			Bilinguals	
	Monolinguals	Mono-dialectals	Bidialectals	Gaelic-English	Asian-English
Age	69.5 (8.6)	69.7 (7.7)	72.4 (8.2)	69.8 (5.5)	72.6 (7.3)
WASI vocabulary	60.3 (9.6)	57.1 (8.4)	55.6 (6.7)	57.9 (9.0)	53.6 (14.2)
WASI matrix	61.0 (10.5)	59.5 (10.3)	59.1 (7.7)	59.5 (5.7)	56.2 (6.9)
Skill level* (1–4)	3.1 (1.2)	2.9 (0.9)	2.4 (0.9)	3.4 (0.8)	2.7 (1.1)
Percentage use of English**	100.0 (0.0)	94.6 (7.3)	52.6 (9.7)	44.3 (15.4)	52.8 (15.0)
Congruent RTs	608.3 (78.6)	576.1 (66.3)	614.9 (173.6)	594.2 (125.8)	714.1 (202.1)
Incongruent RTs	693.7 (125.5)	674.6 (97.6)	696.5 (190.4)	684.5 (157.1)	809 (207.71)
Percentage of error rates	1.8%	1.6%	2.5%	3.1%	4.2%
Simon cost	85.4 (85.4)	98.49 (66.5)	81.6 (51.7)	90.3 (104.0)	94.9 (55.0)

Significant differences between groups based on appropriate statistical test (explanations in text) with *p < .05 and **p < .001.

### Age

Although all participants were over the age of 60 years, it is important to check that there were no age differences between the groups which was confirmed by a one-way analysis of variance (ANOVA), *p* = .62.

### Percent language use

A one-way ANOVA comparing the self-reported percentages of daily use of either Anglo-English or SSE yielded a significant effect of group, *F*(4, 75) = 91.0, *p* < .001. Post-hoc tests using Tamhane's T2 for unequal variances indicated that, as expected, both bilingual groups and the bidialectals reported significantly less use of English (i.e. only an average of 48% of time) than monolinguals and monodialectals, all *p*'s < .001.

### Socio-economic status

We disregarded participant income as a measure of SES as 75% of the participants were retired. Instead, we used the 2010 Standard Occupation Classification (UK Office of National Statistics) to categorise participants' occupations into one of four skill levels based in the amount of formal qualifications or work-based training estimated to be necessary to perform each occupational task. These skill levels ranged from 1 (occupations requiring general education) to 4 (professional/managerial occupations requiring degree-level education). A Kruskal–Wallis test revealed a significant difference between groups, *H*(4) = 9.62, *p* = .047, with mean ranks of 29.0, 36.4, 39.6, 46.5 and 51.0 for bidialectals, Asian Language-English bilinguals, monodialectals, monolinguals and Gaelic-English bilinguals, respectively. A post-hoc test using a Bonferroni-corrected Mann–Whitney test showed that the only significant difference was that between bidialectals who had a significantly lower skill level than Gaelic-English bilinguals, *U* = 51, *p* < .005.

### WASI

WASI scores were missing for one monodialectal participant who was unable to complete the session. One-way ANOVAs comparing performance of the groups on the Vocabulary and Matrix subscales separately yielded no significant effects, all *p*'s > .39.

### Simon task

**Figure 1.**
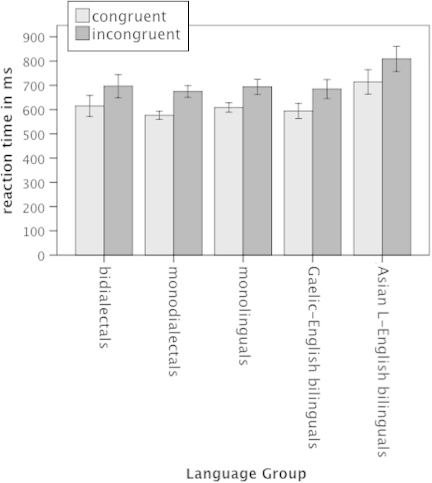
Reaction times for congruent and incongruent trials in the Simon task in bidialectals, monodialectals, monolinguals, Gaelic-English bilinguals and Asian Language-English bilinguals. Error bars show 1 *SE*.

Participants committed a total of 2.7% of errors. Error rates were submitted to a 5 (Group: bidialectal, monodialectal, monolingual, Gaelic-SSE bilingual, Asian Language-English bilingual,) × 2 (Trial Type: congruent, incongruent) ANOVA, which yielded a significant effect of Trial Type: *F*(1, 75) = 24.04, *p* < .001, with incongruent trials yielding higher error rates than congruent trials, but no significant interaction between Trial Type and Language Group. For correct trials, reaction times >2.5 *SD* above the mean were excluded from the analysis, which affected an additional 57 (2.6%) trials. For the RTs (see Figure 1), a similar 5 × 2 ANOVA yielded a main effect of Trial Type, *F*(1, 75) = 115.1, *p* < .001. The effect of Group fell short of significance, *F*(4, 75) = 2.23, *p* = .074, reflecting a trend towards slower RTs in the Asian Language-English bilinguals. There was no significant interaction between Group and Trial Type. An analysis using median RTs yielded similar results showing a main effect of Trial Type, *F*(1, 75) = 81.47, *p* < .001, and an effect of Group which fell short of significance, *F*(4, 75) = 2.43, *p* = .055. Inspection of the distribution of mean and median RTs across all participants revealed one slow outlier in the Asian Language-English bilingual group. Removal of this participant from the reaction time analysis confirmed the effect of Trial Type, *F*(1, 74) = 112.97, *p* < .001, and showed no other effects.

Because higher SES has been associated with improved executive functioning (Morton & Harper, 2007), we conducted an additional ANOVA including Skill Level instead of Group as between-subjects factor. This analysis confirmed the slower reaction time for incongruent trials as indicated by the main effect of Trial Type, *F*(1, 76) = 98.1, *p* < .001, but did not yield any other significant effects.

These results differ from the Bialystok et al.'s (2004) study where older bilinguals exhibited dramatically smaller Simon effects. One possible explanation for this discrepancy may be related to different treatment of reaction time outliers. Bialystok et al. (2004) do not report any exclusion of outliers. To achieve comparability with that study, we repeated the ANOVA with all RTs from the correct responses included. This analysis yielded a main effect of Trial Type, *F*(1, 75) = 10.53, *p* < .01, but no effect of Group and no interaction between the two factors. In sum, as expected, all participants showed significantly slower RTs for incongruent trials in the Simon task confirming that inhibiting the incongruent spatial location of the stimulus required additional inhibitory effort. Crucially, the lack of an interaction suggests that there is no evidence for group differences in the magnitude of the Simon effect, and thus, no evidence for differences in inhibitory control between bilinguals, bidialectals and monolinguals.

However, since traditional null hypothesis significance testing does not directly test acceptance of the null hypothesis (see Kruschke, 2011), we used a Bayesian approach to compare the likelihood of a model assuming no differences in inhibitory control between monolinguals and bilinguals with one assuming that there are differences between monolinguals and bilinguals. To this end, we grouped the three monolingual (*n* = 48) and two bilingual (*n* = 32) groups together and computed the Simon cost as the difference between RTs for congruent and incongruent trials. Using the Bayes Factor (BF) package in R (Morey & Rouder, 2011) and a Cauchy prior (Rouder, Speckman, Sun, Iverson, & Morey, 2009), we obtained BF = 0.179 for the Simon Cost, BF = 0.622 for congruent RTs and BF = 0.541 for incongruent RTs. As it can be argued that our bidialectal participants know two varieties of a language, and hence, it is unclear to what extent they resemble monolinguals or bilinguals, we performed the same analysis with the bidialectal group removed, and obtained BF = 0.189 for the Simon cost, BF = 0.854 for congruent RTs and BF = 0.597 for incongruent RTs. Both of these analyses provide evidence for acceptance of the null hypothesis of no difference between monolinguals and bilinguals in Simon cost as the BFs were below 1 (Rouder et al., 2009).

## DISCUSSION

Our findings did not show an advantage in non-linguistic inhibitory control for older Gaelic-English and Asian Language-English bilinguals, nor did we find such an advantage for bidialectal speakers who routinely use both Dundonian Scots and SSE. Moreover, there was no global reaction time advantage for bilinguals and bidialectals, suggesting that the groups did not differ in general executive processing related to conflict monitoring either (Hilchey & Klein, 2011). If anything, one bilingual group, the Asian Language-English bilinguals showed a trend towards slower RTs, a finding that goes in the opposite direction from what a bilingual advantage would predict.

Our experiment was closely modelled after Experiment 1 in Bialystok et al. (2004). For the monolinguals, that experiment showed mean RTs of 1,437 ms for the congruent trials and 3,150 ms for the incongruent trials. For the bilinguals, the RTs were somewhat faster (congruent: 911 ms, incongruent: 1,959 ms). These are unusually slow overall RTs, in stark contrast to the much faster RTs in our study, which followed the same timing, contained the same number of trials and had a comparable sample size. The magnitude of the Simon effect in Experiment 1 of Bialystok et al. (2004) is also far beyond what is considered to be typical in older adults (Hilchey & Klein, 2011; Van der Lubbe & Verleger, 2002), leaving open the possibility that group differences between older bilinguals and monolinguals emerge only for unusually long RTs which may be indicative of a substantial slowing of cognitive performance in some older populations, perhaps due to diminished experience with computerised testing or due to sub-clinical effects of dementia. However, the fact that subsequent studies (Bialystok et al., 2008; Salvatierra & Rosselli, 2010; Schroeder & Marian, 2012) with larger numbers of trials found a bilingual advantage using the Simon task for older bilinguals displaying overall RTs similar to the ones reported here suggests that the bilingual advantage in Bialystok et al. (2004) is not just an artefact of unusually long RTs.

What then can account for our failure to replicate the findings of Experiment 1 in Bialystok et al. (2004)? We can rule out differences in age and experimental protocol, and we established that differences in SES, cultural and ethnic background as well as immigrant status did not affect the results either. Moreover, typological distance between the languages was large in both studies making this also an unlikely source for the inconsistent results. Potentially, however, there remain qualitative differences in the way in which bilinguals used their two languages throughout their lives as different interactional contexts can impose different demands on executive control processes (Green & Abutalebi, 2013). Compared to dual language use (i.e. use of both languages with different interlocutors in the same setting) dense code-switching and single-language use (i.e. use of both languages in different, non-overlapping settings such as home vs. work) reduce the need for selective response inhibition of the type assessed by the Simon task. Unfortunately, purely quantitative estimates of language use provided in most studies, including ours, make it difficult to identify the habitually encountered interactional contexts for each bilingual. Still, to reconcile the different results, it may be helpful to consider language of schooling as a proxy for habitually encountered interactional context: Schooling in only one language should promote single-language use if the language of schooling is preferred for a wide range of interactional settings and topics, whereas the other language is reserved for a restricted range of settings and topics. Relatedly, if reading is practiced predominantly in one language, it will lead to considerable gains in vocabulary size compared to the other language so that labels for many concepts may be known only in the language preferred for literacy. In such a scenario, lexical competitors from the other language may not be activated for many semantic domains and, hence, are less likely to interfere.

As indicated above, the Gaelic-English bilinguals received no Gaelic-medium schooling during their primary and secondary education, which likely promoted compartmentalised single-language use in many settings, even if overall *amount* of use was balanced. Similarly, most of the Asian Language-English bilinguals immigrated to the UK after having completed their education in their first language, making frequent single-language use also a likely scenario. In contrast, the bilinguals in Bialystok et al. (2004) were educated in both languages allowing for the possibility of more frequent dual-language use. This idea also echoes with the findings from Gaelic-English bilingual children who attended Gaelic-medium schools and showed benefits in verbal and non-verbal IQ compared to Sardinian-Italian bilingual children who received no schooling in Sardinian (Lauchlan, Parisi, & Fadda, 2013). Even though measures of general cognitive ability are different from measures of inhibitory control, this finding may leave open the possibility that schooling in both languages promotes habitual dual-language use and shapes the associated demands on executive control accordingly, which may eventually lead to a bilingual advantage. However, studies on non-immigrant Spanish-Basque bilingual children schooled in both languages showed no cognitive control advantages compared to Spanish monolinguals carefully matched on a variety of variables (Antón et al., 2014; Duñabeitia et al., 2013), although it should be noted that the bilingual children were exposed to Spanish somewhat more frequently. Similarly, Welsh-English bilingual children and adults showed no advantage in cognitive control and meta-linguistic awareness compared to matched English monolinguals regardless of patterns of language dominance and use in the home (Gathercole et al., 2014), although it remains unclear to what extent the bilingual children were schooled in Welsh. These group differences underscore the importance to carefully match the various circumstances that may influence interactional contexts across bilingual populations.

In sum, our failure to observe superior inhibitory control in older Gaelic-English bilinguals, Asian Language-English bilinguals and Dundonian-SSE bidialectals suggests that neither differences in cultural and ethnic background related to immigrant status of the bilinguals, nor routine use of dialect varieties in the monolinguals seem to account for the inconsistencies in findings. Although an analysis of other age groups is beyond the scope of this paper, it is worth mentioning that such inconsistencies in terms of finding a bilingual advantage also exist in studies of inhibitory control using the Simon task in children, in younger and in middle-aged adults, as well as other executive processing tasks (e.g. Stroop task, Flanker task, anti-saccade task) and for other executive processing components (e.g. response suppression, switching, monitoring and updating—for an overview over studies that do not replicate a bilingual advantage see Paap & Greenberg, 2013). To reconcile these inconsistencies, future research needs to describe the specific behavioural ecology of bilingual language use in more detail and develop instruments to establish the recurrent, habitually encountered interactional contexts with the aim of exploring how these contexts shape cognitive control demands and whether they provide the necessary extended practice of inhibition and executive control that may transfer to non-linguistic domains.
